# Improved rock phosphate dissolution from organic acids is driven by nitrate assimilation of bacteria isolated from nitrate and CaCO_3_-rich soil

**DOI:** 10.1371/journal.pone.0283437

**Published:** 2023-03-24

**Authors:** Mercedes Garcia-Sanchez, Isabelle Bertrand, Abdellatif Barakat, Youssef Zeroual, Abdallah Oukarroum, Claude Plassard

**Affiliations:** 1 Eco&Sols, Université de Montpellier, CIRAD, INRAE, IRD, Montpellier SupAgro, Montpellier, France; 2 IATE, Université de Montpellier, CIRAD, INRAE, IRD, Montpellier SupAgro, Montpellier, France; 3 Mohammed VI Polytechnic University (UM6P), Hay Moulay Rachid, Ben Guerir, Morocco; 4 OCP/Situation Innovation, OCP Group, Jorf Lasfar Industrial Complex, Krakcha, Morocco; ICAR-Indian Insitute of Soil Science, Bhopal, INDIA

## Abstract

Until now, the solubilization capacities of insoluble mineral P by soil microorganisms have been screened *in vitro* with media containing NH_4_^+^ as a nitrogen source. This presence of NH_4_^+^ will lead to an acidification of the medium responsible for the solubilization of the insoluble P. However, besides proton release, the production of organic acids can play a very important role in the release of free P. This physiological mechanism can largely depend on the source of nitrogen (NH_4_^+^vs NO_3_^-^) assimilated by the bacteria but the influence of the N source on the production of organic acids has yet to be studied. Our aim was to investigate if the N source assimilated by bacteria and the soil characteristics such as the dominant N source (NH_4_^+^vs NO_3_^-^) and CaCO_3_ contents might influence the bacterial capacities to solubilize rock phosphate. To fill this objective, we screened the capacity of bacteria isolated from 3 soils to solubilize rock phosphate *in vitro* in presence of NH_4_^+^or NO_3_^-^. Then, we selected the most efficient bacterial strains to identify and quantify the release of organic anions into the medium. Among the two hundred and forty-three bacterial strains isolated from the 3 soils, nine and seven isolates were identified with the highest % rock phosphate-solubilization values with NH_4_^+^ or NO_3_^-^ as the sole N-source. Only one strain was able to release free Pi with NH_4_^+^ or NO_3_^-^ as the sole N-source. The most predominant organic acids released by almost all isolates were gluconic acid, lactic acid, glycolic acid, acetic acid, formic acid and pyruvic acid regardless the N-source. However, with NO_3_^-^ as source of N, the highest concentrations on those acids were found together with the highest release of free Pi into the medium. Molecular analysis of 16S rRNA indicated that almost all strains belonged to *Bacillus* and *Paenibacillus* genera. The PCA analysis between soil properties and bacterial capacities to release organic acids and free Pi also revealed that soil factors such as CaCO_3_ and soil NO_3_^-^ content positively influenced the release of organic acids by bacteria grown *in vitro*. Our results concluded that the bacterial rock phosphate-solubilization was intimately related to organic acids production which in turn seemed to be driven by the assimilation of NO_3_^-^ by bacteria. Therefore, the N-source might be considered a key factor to take into consideration during the screening and selection of suitable strains involved in the P-solubilization.

## Introduction

Phosphorus (P) is essential for life and is one of the key macro-nutrients for plant growth and development, including photosynthesis, energy transfers and nutrient uptake in plants [1–3]. Although organic and inorganic P forms are abundant in soils, they are highly insoluble (95–99%) with little mobility and low availability in most soil conditions [4]. Therefore, P availability is often limiting plant growth due to its poor use efficiency (5–30%). Unfortunately, even when using high rates of chemical P-fertilizers application a considerable amount of the applied P-based chemical fertilizer is rapidly immobilized and precipitated with cations such as Ca^2+^, Al^3+^ and Fe^3+^, which lead to the formation of poorly available P for plants [5]. Besides, excessive application of P causes environmental and economic problems due to soil erosion and runoff water containing large amount of soluble P [6]. The application of raw forms of P such as rock phosphate (RP), which is the source of P fertilizers, has been extensively studied as a method to overcome the problems of P limitation in agronomic crops [7]. Rock phosphates are less expensive than purified and formulated P fertilizers, but are insoluble especially in soils with high pH and low organic matter, limiting its direct use as soil amendment [8]. Faced with this situation, the idea arises to implement low technological cost alternatives to improve the acquisition of P by plants from RP; these include the use of rhizosphere and endophyte microorganisms that promote the solubilization of P from chemically forms such as RP [9–11].

Phosphate-solubilizing bacteria (PSB) play a fundamental role in biogeochemical P cycling in agricultural ecosystems, as they are involved in the transformation of insoluble forms of P into monobasic and dibasic phosphate (HPO_4_^2-^, H_2_PO_4_^-^) available to plants [12]. The better understanding of the mechanisms responsible of RP solubilization takes a crucial role about how to improve the efficiency of PSB [13]. Although several mechanisms may be responsible of P solubilization, the main one is through the production of organic acids which involves either: (i) acidification by lowering pH, (ii) chelation of cations (mainly Ca^2+^) bound to phosphate through their hydroxyl and carboxyl groups, (iii) exchange reactions with phosphate for adsorption sites or (iv) formation of soluble complexes with metals [14–16]. Proton-excretion accompanying NH_4_^+^ assimilation is thought to be an alternative mechanism of P solubilization which has been found in some bacterial species, i.e. *Pseudomonas fluorescens* RAF15 or *Bacillus marisflavi* FA7 [6, 17]. At present, a key knowledge gap is the role that N supply plays in the mechanisms of RP solubilization since PSB need N and organic C to produce organic acids that solubilize the P. Besides, the production of organic acids requires N for transcription and translation [18]. Since N addition is required for the production of solubilizing compounds, the N-source could control the extent of acid production by PSB which would be crucial to evaluate the suitability of PSB as effective RP solubilizer in the plant rhizosphere or for their use in the biotechnological production of RP-based fertilizers.

In this work, we firstly hypothesized that P solubilization of RP depends on the release of organic acids by PSB strains and is greatly influenced by the N source (NO_3_^-^ and NH_4_^+^) supplied to the bacteria. We secondly hypothesized that the physical-chemical properties of soils such as texture, pH, available-P, N-NH_4_^+^, N-NO_3_^-^ and CaCO_3_ might also affect the bacterial features in terms of RP solubilization. To test the first and second hypotheses, we used a collection of two hundred and forty-three strains isolated from three contrasted soils. The isolates were at first screened using the National Botanical Research Institute´s phosphate NBRIP agarose media with NH_4_^+^ or NO_3_^-^ as the sole N-source. Then, their capacity to release soluble-P from RP was evaluated to select the strains with the highest percentage of RP-solubilization. The effect of the two N-sources on the bacterial growth, pH changes and organic acid production in the culture medium associated with the capacity to solubilize the RP was also reported. Thirdly, the isolates with the highest capacity to solubilize the RP were identified based on sequencing and phylogenetic analysis of the 16S rRNA gene.

## Material and methods

### Soil sampling

Three sites of sampling, differing in their physico-chemicals properties, were selected to isolate rock phosphate solubilizing bacteria. Soils were identified as “A”, “B”, and “C” (Table 1) and were collected from the top soil (0–20 cm depth) of cropped fields. The soil A was collected from the DIASCOPE INRAE experimental station located in the South of France (Mauguio) (43.612°N; 3.976°E). It was classified as Skeletic Rhodic Luvisols [19], and was characterized by its neutral to alkaline pH and high stones content. The soil B, was sampled from the experimental site of Restinclières located at Prades-le-lez, 15km North of Montpellier (43°42′15 N, 3°51′41 E) and was classified as a deep Fluvisol (WRB, 2007) with high level of CaCO3 leading to alkaline pH [20]. The soil C, was sampled from the “Faidherbi-Flux” collaborative observatory for greenhouse gas balance and ecosystem services (https://lped.info/wikiObsSN/?Faidherbia-Flux). It is located in the natural agro-silvo-pastoral parkland of Sob (14◦29′45N, 16◦27′13W), 135 km East of Dakar, West Senegal [21]. It is classified as an Arenosol [19] with very low level of C and P. All soil samples were collected from three pseudo replicates then mixed and immediately homogenized, sieved through a 2-mm diameter mesh, and stored at 4 ºC until analysis.

**Table 1:** Physical-chemical characteristics of the collected soil samples used for the isolation of rock phosphates-solubilizing bacteria. Data are given as means ± standard deviation (n = 3).

	Soil A	Soil B	Soil C
Clay (< 2 μm) (g kg^-1^)	210±4.4	177±5.2	50±17
Fine silt (2–20 μm) (g kg^-1^)	121±9.5	83±4.5	20.3±2.1
Coarse silt (20–50 μm) (g kg^-1^)	194.3±8.5	83±2.3	60.2±7
Fine sand (20–200 μm) (g kg^-1^)	203.6±10.1	83±5	547±18
Coarse sand (200–2000 μm) (g kg^-1^)	270.3±10.4	19.8±1.8	321±23
CaCO_3_ (g kg^-1^)	1±0	534.3±2.3	n.d
Total C (g kg^-1^)	10±0.7	19.5±1.40	5±1
Total N (g kg^-1^)	1±0.05	1.6±0.9	0.4±0.1
N-NO_3_^-^ (mg kg^-1^)	4.6±1.81	15±1.4	8.9±3
N-NH_4_^+^ (mg kg^-1^)	7.7±0.3	3±0.25	0.5±0.2
Olsen P (mg kg^-1^)	40±6	20±2	20±2
pH	7.5±0.2	8.4±0.01	7±0.1
Sampling site	Mauguio (France)	Restinclières (France)	Dakar (Senegal)

### Rock phosphate

The rock phosphate (RP) used in this study was supplied by the OCP (Office Chérifien des Phosphates), Ben Guerir, Morocco. The analysis for the total contents of the elements on its composition was performed through inductively coupled plasma (ICP) at the laboratory of Arras (France) (S1 Table).

### Isolation of bacterial strains

For bacterial isolation, 1 g of soil was dispersed in 100 mL of 1% NaCl in 250 mL Erlenmeyer flasks by shaking in an orbital shaker. Subsequently, several serial dilutions (10^−2^–10^−7^) were prepared in NaCl and 0.1 mL of these dilutions were spread with sterile glass rods in Petri dishes filled with solidified CASO agar (Sigma) medium (containing 15 g L^-1^ casein peptone, 5 g L^-1^ soy peptone and 15 g L^-1^ agar). The Petri plates were incubated for 4 weeks at 18 ºC in the dark. After incubation, the plates with the lowest dilutions and the least numbers of colonies were chosen for bacterial isolation. Subsequently, starting from the most dilute plates, the first 200 colonies encountered were transferred to new Petri dishes (60 mm) with CASO agar media and were incubated in the dark at 18 ºC for three weeks. From each soil sample, unpurified and non-growing colonies were discarded. Afterwards, bacterial colonies of each soil sample (A, B and C) were chosen at random. Each strain was numbered, and 93, 100, and 50 numbers were randomly selected for soil samples A, B, and C, respectively. In total, 243 isolates were further evaluated on their abilities to solubilize the rock phosphate.

### Screening of RP-solubilization bacteria with different N-sources

A *«spot test»* was used to screen rapidly the ability of bacterial strains previously isolated to solubilize the rock phosphate according to the N-source, that was either NH_4_^+^ or NO_3_^-^. The screening was run on agarose square Petri dishes (12 × 12 cm) which were filled with 70 mL of National Botanical Research Institute´s phosphate (NBRIP) medium [22] with some modifications and containing, per liter: 10 g glucose, 5 g MgCl_6_ *6 H_2_O, 0.25 g MgSO_4_*7H_2_O, 0.2 g KCl, 2.5 g rock phosphate, 0.1 g (NH_4_)_2_SO_4_ or KNO_3_, 1 mL of a vitamin solution (containing, per liter: 5 g panthotenate, 20 g inositol, 2 g nicotinic acid, 0.25 g pyridoxal hydrochloride, 0.25 g thiamine hydrochloride, and 0.01 g D-biotine), 0.006 g bromophenol blue (pH indicator dye), and 18 g agarose. A pre-inoculum of each bacterial strain was prepared in a 96-well plate containing LB medium and incubated for 3 days at 28 ºC. After that, 10 μL of each bacterial suspension was inoculated on NBRIP agarose medium with RP and NH_4_^+^ or NO_3_^-^ as the sole source of P and N, respectively. The plates were then incubated at 28 ºC till the occurrence of a halo of solubilization (yellow area around the colony) appearing generally after 7 days of incubation. Hence, all colonies were checked at 7 days, and only those producing halos were selected.

### Bacterial growth, RP solubilisation capacity and production of organic acids in liquid NBRIP media with different N-sources

Three experiments were conducted to test the capacity of the isolates to solubilize the RP by organic acids production with different N-sources. The first experiment was conducted to measure quantitatively the percentage of the RP solubilization (% RP-solubilization value) on the previous selected colonies to identify the isolates with the highest capacities to release the soluble-P for further experiments. To do this, 200 μL of each bacterial culture previously grown in LB media for 72 h at 28°C (exponential phase) were used to inoculate 50 mL of NBRIP liquid media with the same composition as above-mentioned. The flask containing the inoculated media was incubated for 7 days at 28 ºC. A non-inoculated treatment was also set up. At 7 days, 1 mL of the NBRIP liquid medium was collected and centrifuged for 10 min at 3,100 rcf to obtain a free-bacterial NBRIP medium to measure the soluble-P content using the malachite green method [23]. The % RP-solubilization value of each bacterial isolate was then calculated as follow: (soluble-P _isolate_/P total), with soluble-P _isolate_ as the soluble-P content (μg mL^-1^) release from the RP into the NBRIP liquid medium, and P total indicated the total P added in the NBRIP liquid media as RP (2.5 g L^-1^). Since a residual soluble-P content release from the RP was expected as the result of the incubation, the % RP-solubilization value was also calculated for the non-inoculated treatments. The experiment was run in triplicate.

The second experiment was set up to evaluate the bacterial growth and RP-solubilization capacity on the above-selected isolates after 0, 3, 7, 14 and 21 days of culture. 1 mL of the bacterial suspensions, produced as before, was used to measure the optical density (OD) at 600 nm as an indirect measurement of bacterial growth using a UV-Visible Spectrometer (Spectronis Helios α UV-Vis). Afterwards, the bacterial suspension was subjected to a centrifugation for 10 min at 3,100 rcf to obtain a free-bacterial NBRIP medium which was used to measure the pH with a pH microelectrode (Fisherbrand^™^, diameter of 3 mm), and the soluble-P content using the malachite green method.

The third experiment was performed to identify and to quantify the production of organic acids in the free-bacterial NBRIP liquid medium of each bacterial strain after 21 days of incubation. The free-bacterial NBRIP liquid medium with NH_4_^+^ as the sole N-source contained salts such as MgCl_2_*6H_2_O, KCl, (NH_4_)_2_SO_4_ and MgSO_4_*7H_2_O, resulting in high amounts of Cl^-^ and SO_4_^=^ ions incompatible with HPIC analysis. To remove these ions, the free-bacterial NBRIP liquid media were filtrated using cartridges in series containing Ag (Dionex OnGuard II-AG cartridge, Thermo Scientific) and Ba (Dionex OnGuard II-Ba cartridge, Thermo Scientific), trapping Cl^-^ and SO_4_^=^, respectively. Meanwhile, the free-bacterial NBRIP liquid media with NO_3_^-^ as the sole N-source contained high amounts of salts such as MgCl_2_*6H_2_O and KCl, resulting in elevated Cl^-^ concentrations. In this case, Cl^-^ ions were eliminated using only cartridges containing Ag (Dionex OnGuard II-AG cartridge, Thermo Scientific). The cartridges were rinsed with 10 ml ultrapure water before filtering 1 ml of each free-bacterial NBRIP liquid medium through the cartridge. The two free-bacterial NBRIP liquid media with NH_4_^+^ or NO_3_^-^ were also passed through a 0.45 μm filter and diluted with ultrapure water before to be analyzed in an HPIC system (Dionex IonPac) equipped with an AS11-HC-11 μm column (4 × 250 mm) and a Dionex AERS 500 suppressor. All separations were performed at a flow rate of 1 ml min^−1^. We tested various KOH gradients in the mobile phase to improve the separation of organic anions from mineral ones. Finally, the gradient composition we used for our analyses is given in S2 Table. The column was at room temperature (25 ± 2 °C). The injection volume was 25 μl taken from the sample prepared in a 1 ml vial. Solutions of 15 organic anions were first prepared from the synthetic compounds listed in Table 4. For each compound, different solutions were prepared in ultrapure water that were (1) a stock solution, kept at 4°C; (2) a solution for determining the retention time (RT) of the organic anion and (3) solutions with 7 final concentrations for building a calibration curve (S3 Table). Each sample of free-bacterial NBRIP medium was injected 3 times (100 μL per injection). Peaks on each chromatogram were compared with the chromatogram obtained with standard solution to identify each organic acid, and concentrations were extrapolated from the standard curve.

### 16S rRNA gene sequencing from bacterial isolates

The taxonomic identities of bacterial strains showing the highest ability to solubilize the RP with NH_4_^+^ or NO_3_^-^ were assigned by 16S rRNA gene sequence analysis. Bacterial genomic DNA was extracted by suspending a few colonies from each strain in 1 mL of MilliQ water in a microcentrifuge tube. For nearly full-length amplification of the 16S rRNA, the primer pair FD1 (5ʹ -AGAGTTTGATCCTGGCTCAG- 3ʹ) and RP2 (5ʹ- ACGGCTACCTTGTTACGACTT-3ʹ) was used [24]. PCR mixtures were composed of 5 μL PCR buffer (10x), 4 μL MgCl_2_ (final concentration 2 mM), 1 μL dNTPs (10 mM each), 1 μL each forward and reverse primers (10 mM), 0.3 μL Taq polymerase (5 U μL^-1^ Qbiogene) and 5 μL DNA solution in a total volume of 50 μL. The thermal cycling program was: i) 5 min at 95 ºC, ii) 35 cycles: 15 s at 94 ºC, 30 s at 60 ºC, and 90 min at 72 ºC, iii) 10 min at 72 ºC. PCR products were purified and sequenced using the internal primers 926F (5ʹ -AAACTYAAAKGAATTGACGG-3 ʹ) and 1100R (5ʹ -GGGTTGCGCTCGTTG- 3ʹ) at Genewiz (Leipzig, Germany). The sequence obtained for each isolate was compared for similarity level with the reference strains from genomic database banks using Rdp taxonomy tool available at the https://www.rdp.cme.msu.edu web site. The sequences were registered in NCBI under the number given in Table 5. The phylogenetic tree was built using phylogenia.fr [25–31].

### Data analysis

Unless otherwise stated, the results are given as mean ± standard error (n = 3). Statistical analysis was carried out using Statistica 13 software. The normality was firstly tested using a Levene test. When normality was reached, the ability of isolates to solubilize the RP (soluble-P content and % RP-solubilization ability) with NH_4_^+^ or NO_3_^-^ as the sole N-source after 7 days of culture was analyzed using one-way ANOVA to select the most effectives isolates. The same was performed on the organic acids produced by the bacterial strains after 21 days of incubation. Comparison of means was performed by Tukey´s HSD post hoc at three levels of significance: *p<0.05; **p<0.01; ***p<0.001.

Two-way ANOVA with repeated measures was run to evaluate significant differences among bacterial strains and time of incubation on bacterial growth (OD 600 nm), soluble-P content and pH. To gain a better understanding about the distribution of the physical-chemical properties (clay, fine silt, coarse silt, fine sand, coarse sand, CaCO_3_, total C, total N, N-NO_3_^-^, N-NH_4_^+^, Olsen P and pH) in the three soil samples collected (A, B and C), a principal component analysis (PCA) was done using the CANOCO 5.0 for windows. To gain a new knowledge about the influence of the N source in the production of organic acids and thus, in the RP-solubilization, an integrated multivariate analysis was also run, using the same software, to determinate a new set of correlations among: i) the RP-solubilization isolates from soil A, B and C with NH_4_^+^ or NO_3_^-^ as the sole N-source, ii) the soluble-P content and the organic acids: gluconic, lactic, glycolic, acetic, butyric, formic, propionic, pyruvic, malic, maleic, oxalic and citric acids measured in the NBRIP liquid medium after 21 days of culture, and iii) the physical-chemical soil properties from soil A (Olsen P, N-NH_4_^+^ content and coarse silt), B (N-NO3- and CaCO_3_ content) and C (coarse and fine sand) selected through the first PCA analysis. These statistical analyses were run on reduced and centered variables.

## Results

### Screening of RP-solubilization bacteria under different N sources

A total of two hundred and forty-three bacterial colonies, isolated from soils A, B and C, were screened on their abilities to solubilize the RP using the *«spot test»* on NBRIP agarose media with NH_4_^+^ or NO_3_^-^ as the sole N-source. In total, forty isolates were able to solubilize the RP due to the presence of halo with NH_4_^+^ or NO_3_^-^. Twenty-three isolates out of these forty strains had the capabilities to solubilize the RP (presence of solubilization halo) with NH_4_^+^, while seventeen isolates out of forty strains produced clear halos in NBRIP agarose media with NO_3_^-^. Remarkably, none of the isolate had same ability to solubilize the RP with either NH_4_^+^ or NO_3_^-^ as N-source, except one isolate displaying a halo on both N-sources.

### Bacterial growth and RP solubilization capacity under different N-sources

A first set of experiments was established to evaluate the capacity of the bacterial strains, producing a halo of solubilization, to release the soluble-P content from RP and thus, measure the % RP-solubilization value with NH_4_^+^ or NO_3_^-^ as the sole N-source, after 7 days of culture (Table 2). A residual amount of soluble-P was detected in each non-inoculated NBRIP liquid media (NI) with NH_4_^+^ or NO_3_^-^ at 7 days, reaching values between 8.2–9.6 μg mL^-1^, respectively. When NH_4_^+^ was supplied as the sole N-source, only nine isolates, identified as 59B, 24A, 4A, 9C, 87B, 12A, 47A, 15A and 46B out of twenty-three strains significantly increased the % RP-solubilization values ranging from 3.5 to 9.2% after 7 days of incubation. Meanwhile, with NO_3_^-^ as the sole N-source, seven strains, labeled as 23B, 48B, 41C, 39B, 59B, 32A and 6C out of seventeen isolates strongly release the soluble-P from the RP with % RP-solubilization values ranging between 2.4–36% (Table 2). The isolate 59B, which was able to solubilize the RP with NH_4_^+^ or NO_3_^-^, showed higher % RP-solubilization with NH_4_^+^ (9%) compared to NO_3_^-^ (3%). The isolates with the highest % RP-solubilization were then selected for the experiment described below.

**Table 2:** Soluble-P content and RP-solubilization ability (RP solub.) of bacterial strains in NBRIP liquid media with NH_4_^+^ or NO_3_^-^ as the sole N source after 7 days of culture. Data are given as means ± standard error (n = 3). The same lowercase letters in each column indicate a lack of significance (p<0.05) among the different bacterial strains.

N Source					
NH_4_^+^			NO_3_^-^		
Bacterial strains	Soluble-P (μg mL^-1^)	RP solub. (%)	Bacterial strains	Soluble-P (μg mL^-1^)	RP solub. (%)
59B	32.6 ± 11.9^d^	9.20^d^	23B	98.4 ± 0.7^e^	36.0^e^
24A	25.1 ± 1.4^c^	6.19^c^	48B	40.1 ± 6.1^d^	12.76^d^
4A	24.9 ± 4.1^c^	6.10^c^	41C	26.5 ± 6.5^c^	7.32^c^
9C	23.1 ± 5^c^	5.41^c^	39B	24.15 ± 13.4^c^	6.37^c^
87B	22.5 ± 1.8^c^	5.16^c^	59B	17 ± 2.9^b^	3.52^b^
12A	22 ± 2.4^c^	4.97^c^	32A	16.6 ± 0.7^b^	3.33^b^
47A	19.2 ± 7.4^b^	3.85^b^	6C	14.3 ± 1.2^b^	2.42^b^
15A	18.8 ± 4.6^b^	3.66^b^	40B	11.3 ± 3.1^a^	1.22^a^
46B	18.3 ± 1.1^b^	3.47^b^	54B	8.7 ± 1^a^	0.19^a^
46A	14.9 ± 2.2^a^	2.14^a^	8C	7.9 ± 0.7^a^	0^a^
104B	14.4 ± 0.8^a^	1.90^a^	7C	7.7 ± 1^a^	0^a^
67B	12.7 ± 0.5^a^	1.23^a^	4C	7.3 ± 1.8^a^	0^a^
15B	12.5 ± 0.5^a^	1.15^a^	22A	7.2 ± 1.4^a^	0^a^
31B	12 ± 1.3^a^	0.96^a^	30A	6.4 ± 0.5^a^	0^a^
85A	11.7 ± 2^a^	0.82^a^	125B	5.7 ± 0.8^a^	0^a^
4C	11.7 ± 2.8^a^	0.83^a^	29C	5.7 ± 0.1^a^	0^a^
11B	11.2 ± 0.07^a^	0.63ª	2A	5.1 ± 0.4^a^	0^a^
21C	11.1 ± 3^a^	0.60^a^	*NI	8.2 ± 0.5^a^	-
107B	10.5 ± 1.4^a^	0.36^a^			
44A	10.4 ± 1^a^	0.31^a^			
1C	10.3 ± 1.4^a^	0.26^a^			
1B	10.1 ± 2.5^a^	0.18^a^			
27A	8.6 ± 1.3^a^	0^a^			
*NI	9.6 ± 3.2^a^	-			

*NI-non-inoculated NBRIP liquid media

**Fig 1:**
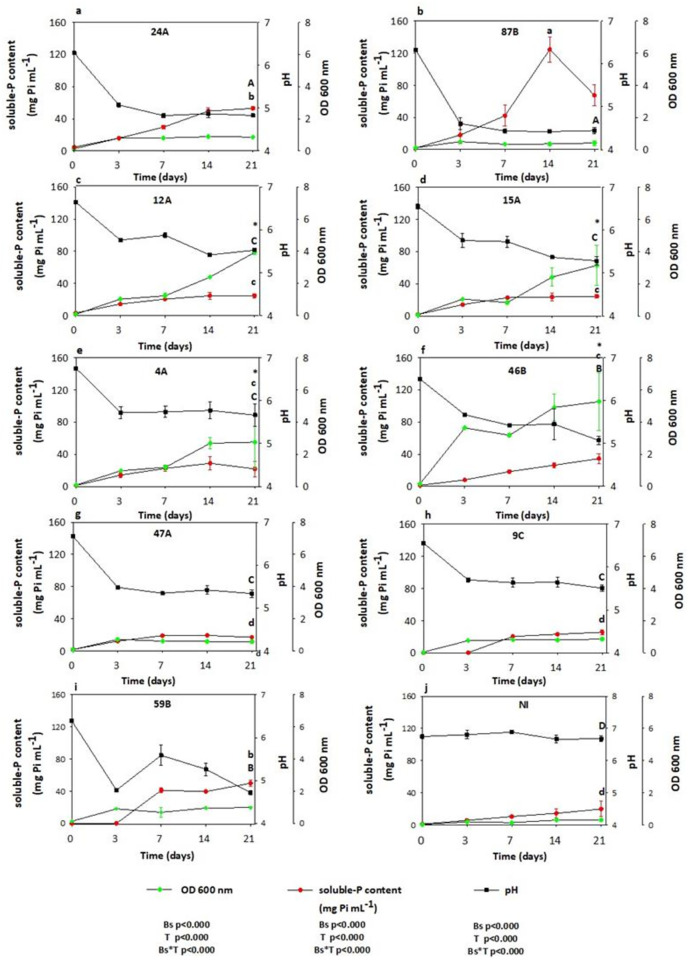
Bacterial growth, soluble-P content (μg Pi mL^-1^) and pH of each isolate cultivated in NBRIP with rock phosphate (RP) and NH_4_^+^ as the sole source of N after 0, 3, 7, 14, and 21 days of culture. Values represent the means and the standard error (n = 3). The effect of bacterial strains (Bs), time of incubation (T) and their interactions (Bs*T) were analyzed with a two-way repeated ANOVA for each variable measured (OD 600nm, soluble-P content and pH). The asterisks indicate the strains with the highest bacterial growth (OD 600nm) over the time of culture. The lower and uppercase letters denote statistical differences in relation to soluble-P content and pH values, respectively, among the isolates after 21 days of experiment.

**Fig 2:**
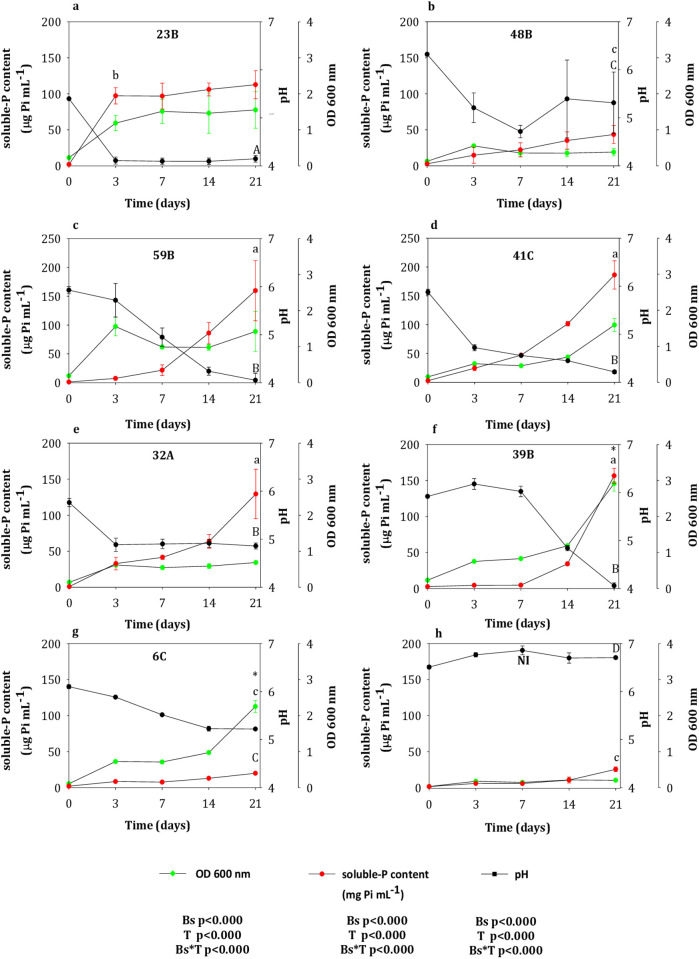
Bacterial growth, soluble-P content (μg Pi mL^-1^) and pH of each isolate cultivated in NBRIP with rock phosphate (RP) and NO_3_^-^ as the sole source of N after 0, 3, 7, 14, and 21 days of culture. Values represent the means and the standard error (n = 3). The effect of bacterial strains (Bs), time of incubation (T) and their interactions (Bs*T) were analyzed with a two-way repeated ANOVA for each variable measured (OD 600nm, solble-P content and pH). The asterisks indicate the strains with the highest bacterial growth (OD 600nm) over the time of culture. The lower and uppercase letters denote statistical differences in relation to soluble-P content and pH values, respectively, among the isolates at any time of the experiment.

The second set of experiments was set up to evaluate the bacterial growth (OD 600 nm), soluble-P content and pH values of the above-selected isolates inoculated in the NBRIP liquid media with RP and NH_4_^+^ or NO_3_^-^ as the sole source of P and N after 3, 7, 14 and 21 days of culture (Figs 1 and 2). When NH_4_^+^ was added as the sole N-source, the OD detected in the non-inoculated NBRIP liquid media reached values up to 0.3 as the result of the slow release of soluble-P content from RP after 21 days of incubation (Fig 1j). However, all isolates were found to increase gradually the OD over the time of culture, becoming higher than the non-inoculated NBRIP liquid media (Fig 1a–1i). The highest bacterial growth was found for 12A, 15A, 4A and 46B isolates which significantly differ compared to the other strains at the end of the experiment (Fig 1c–1f) (Bs, p≤0.000; T, p≤0.000). The residual soluble-P content detected in the non-inoculated NBRIP liquid media reached values up to 20 μg mL^-1^ after 21 days of incubation with steady pH values during the experiment (Fig 1j). However, all bacterial strains were capable to release the soluble-P from RP over the time of incubation with a concomitant pH drop after 3 days of culture (soluble-P content, Bs, p≤0.000; T, p≤0.000; pH, Bs, p≤0.000; T, p≤0.000) (Fig 1a–1i). The isolates could be separated in two groups, the first one including three bacterial strains (24A, 87B and 59B) able to release soluble-P amounts higher than 50 μg mL^-1^ and to decrease concomitantly the pH value of the medium below 5. Among these strains, the 87B one was the most efficient to release soluble-P, with values ranging from 124 (day 14) to 50 (day 21) μg mL^-1^ and a final pH medium of 4.4 (Fig 1b). The two other isolates (24A and 59B) released also soluble-P in their medium with concentrations increasing regularly with the culture time, reaching 50 μg mL^-1^ after 21 days. Both isolates acidified their culture medium, with pH values about 4.8 (Fig 1a and 1i). The second group includes the 6 other isolates tested, namely 12A, 15A, 4A, 46B, 47A and 9C. The isolate 47A was less efficient than the other ones to release soluble-P amounting to 24 μg mL^-1^, despite its ability to decrease the medium pH down to 5.5 (Fig 1g). All the other isolates were able to release similar amounts of soluble-P, with values ranging from 24 to 36 μg mL^-1^ (Fig 1c–1f and 1h).

As previously found in NH_4_^+^ medium, the OD detected in the non-inoculated NBRIP liquid medium with NO_3_^-^ as the sole N-source reached values up to 0.2 (Fig 2j). Even though all isolates increased gradually their growth over the time, the highest OD values were found for 39B and 6C isolates, in contrast with the lowest growth, which was found for 48B strain after 21 days of culture (Fig 2b, 2f and 2g) (Bs, p≤0.000; T, p≤0.000). A residual amount of soluble-P was detected in the non-inoculated NBRIP liquid medium reaching values up to 25 μg mL^-1^ after 21 days of incubation with no changes in the pH values during the experiment (Fig 2h). Overall, all isolates tested significantly increased the release of the soluble-P from RP over the time of experiment with a concomitant pH drop recorded at 3 days, which in some cases, then slowly declined until 21 days of culture (soluble-P content, Bs, p≤0.000; T, p≤0.000; pH, p≤0.000; T, p≤0.000) (Fig 2a–2h). Among the seven strains tested, five of them were able to release high amounts of soluble-P with different kinetics. For example, isolate 23B was detected as the fastest isolate to release soluble-P with values up to 97 μg P mL^-1^ at 3 days, which were also related to a pH drop (4.1) (Fig 2a). In contrast, at 21 days, 59B, 41C, 32A, and 39B isolates were more efficient in releasing soluble-P from RP reaching values up to 159, 186, 129, and 156 μg P mL^-1^, respectively, with pH values ranging from 4 to 4.8 (Fig 2c–2f). Finally, the two remaining strains (48B and 6C) were found to be the less efficient ones in solubilizing the RP over the time of experiment although both strains were also able to reduce significantly the pH of the medium (Fig 2b and 2g).

### Identification and quantification of organic acids

The third set of experiments was run to identify and to quantify the organic acids produced by the selected bacterial strains with RP and N-sources (NH_4_^+^ versus NO_3_^-^) as the sole source of P and N after 21 days of culture (Tables 3 and 4). The HPIC analysis revealed that with RP and NH_4_^+^ as the sole source of P and N, the isolates were able to release eight organic acids (gluconic, lactic, glycolic, acetic, propionic, formic, pyruvic and maleic acids) after 21 days of incubation (Table 3). Among these organic acids, only three of them (gluconic, lactic and pyruvic acids) were released by all strains. Gluconic and lactic acids were produced at the highest rates, with gluconic acid concentrations reaching up to 9 and 11 mM released by isolates 12A and 47A, respectively, and lactic acid up to 7 mM released by isolates 47A and 87B. Pyruvic acid was released at rates ranging between 0.7 (isolate 87B) up to 5.7 mM (isolate 12A). Secretion of formic acid was released by all strains but 15A and 4A and was highly heterogeneous, with concentrations varying from 0.09 (isolate 12A) up to 8 mM (isolate 87B). Only three strains were able to produce propionic acid (12A, 47A and 9C) or maleic acid (59B, 4A and 9C). The strain 9C released the highest concentrations of propionic (1.6 mM) and maleic (2.6 mM) acids. Glycolic acid was found in the culture medium of five isolates at low concentrations, ranging from 0.004 (isolate 87B) to 0.2 mM (isolate 4A). Finally, acetic acid was only produced by 24A with values up to 3.3 mM.

**Table 3:** Concentrations of organic acids (mM) produced by different bacterial strains grown in NBRIP liquid media with rock phosphate (RP) and NH_4_^+^ as the sole source of P and N. Data are given as means ± standard error (n = 3). The same lowercase letters in each row indicate a lack of significance (p<0.05) among the different bacterial strains within each organic acids after 21 days of culture.

Organic acids	Bacterial strains								
	59B	24A	47A	9C	87B	12A	4A	15A	46B
Gluconic	1±0.4^b^	0.8±0.2^b^	11.1±2.3^a^	1±0.3^b^	0.6±0.2^b^	9.3±1.8^a^	1.5±0.5^b^	3±1.5^b^	3±1^b^
Lactic	5.2±0.1^ab^	3.2±0.2^bcd^	6.2±0.66^a^	5 ±0.2^abc^	6.9±0.4^a^	2.9±1.8^cd^	0.3±0.2^e^	1.2±0.8^de^	0.3±0.3^e^
Glycolic	0.08±0.04^ab^	nd	0.2±0.1^a^	nd	0.004±0.004^b^	0.1±0.01^ab^	nd	0.03±0.03^b^	nd
Acetic	nd	3.3±1.6^a^	nd	nd	nd	nd	nd	nd	nd
Propionic	nd	nd	nd	1.6±0.1^a^	nd	0.37±0.3^b^	0.07±0.04^bc^	nd	nd
Formic	2±0.8^b^	1.4±0.5^d^	nd	1.5±0.8^c^	8±0.4^a^	0.09±0.09^f^	0.2±0.1^f^	nd	0.4±0.04^e^
Pyruvic	1.1±0.3^fg^	2.4±06^def^	4.2±0.2^bc^	1.7±0.3^efg^	0.7±0.07^g^	5.7±0.7^a^	2.7±0.6^de^	5.2±0.6^ab^	3.7±0.9^cd^
Maleic	0.7±0.4^b^	nd	0.2±0.09^c^	2.6±0.7^a^	nd	nd	nd	nd	nd

**Table 4:** Concentrations of organic acids (mM) produced by different bacterial strains grown in NBRIP liquid media with rock phosphate (RP) and NO_3_^-^ as the sole source of P and N. Data are given as means ± standard error (n = 3). The same lowercase letters in each row indicate a lack of significance (p<0.05) among the different bacterial strains within each organic acids after 21 days of culture.

Organic acids	Bacterial strains						
	23B	48B	41C	39B	59B	32A	6C
Gluconic	nd	0.5±0.06^d^	5.6±03^a^	nd	nd	3.5±0.4^b^	1.5±0.2^c^
Lactic	61.6±6.5^a^	2.1±0.2^c^	17.3±0.6^b^	52±1.3^a^	16.1±5.6^b^	8.7±0.1^bc^	4.3±0.6^c^
Glycolic	nd	0.01±0.02^d^	2.7±0.3^b^	7.2±0.4^a^	nd	1.6±0.2^c^	nd
Acetic	nd	nd	13.2±0.1^b^	nd	2.9±0.8^c^	30.5±2^a^	13.3±0.6^b^
Formic	0.6±0.3^de^	2.3±0.2^d^	19.3±0.05^a^	10.2±0.5^c^	0.1±0.05^e^	15±1.2^b^	9.8±0.8^c^
Butyric	nd	nd	nd	nd	nd	nd	0.04±0.04^a^
Pyruvic	nd	0.7±0.04^c^	1.3±0.2^bc^	2.8±0.6^b^	0.7±0.09^c^	13.5±1.1^a^	1.7±0.005^bc^
Malic	nd	nd	0.6±0.02^a^	nd	nd	0.8±0.1^a^	0.1±0.005^b^
Oxalic	nd	nd	nd	0.50±0.03^a^	nd	nd	nd
Citric	nd	0.25±0.05^b^	1.06±0.3^a^	0.02±0.009^b^	nd	0.02±0.001^b^	0.03±0.02^b^

With NO_3_^-^ as the sole N-source, ten organic acids were identified in the culture medium after 21 days of experiment (Table 4). Half of the organic acids (gluconic, lactic, glycolic, acetic and formic acids) displayed high concentrations in the culture medium, with values generally greater than 1 mM. The gluconic acid was produced by all strains except 23B, 39B and 59B, with the greatest concentration measured for 41C (5.6 mM). Conversely, lactic acid was secreted by all isolates with the highest concentration for 23B and 39B strains reaching values up to 61 and 52 mM, respectively. The majority of the isolates, except 23B, 59B and 6C, were able to secrete glycolic acid with the highest level for 39B (7.2 mM). The 23B, 48B and 39B strains did not secrete acetic acid, but the most efficient isolate was 32A reaching values up to 30 mM. Formic acid was produced by all isolates with the greatest values for 41C (19 mM). The five remaining organic acids (butyric, pyruvic, malic, oxalic and citric acids) were found at much lower concentrations than the previous ones, with values generally in the mM range. The 6C strain was found the only isolate with abilities to secrete butyric acid, but at very low concentration (0.04 mM). All bacterial strains were able to produce pyruvic acid except the 23B isolate. The greatest concentrations were found for 32A with values up to 13 mM. Malic acid was produced by 41C and 32A at greater rates than 6C, reaching values between 0.8 and 0.6 mM, respectively. Oxalic acid was only produced by 39B with concentrations up to 0.50 mM. Citric acid was secreted by all strains, except 23B and 59B. The greatest concentrations were found for 41C reaching values up to 1 mM.

### Identification of the bacterial isolates

**Fig 3:**
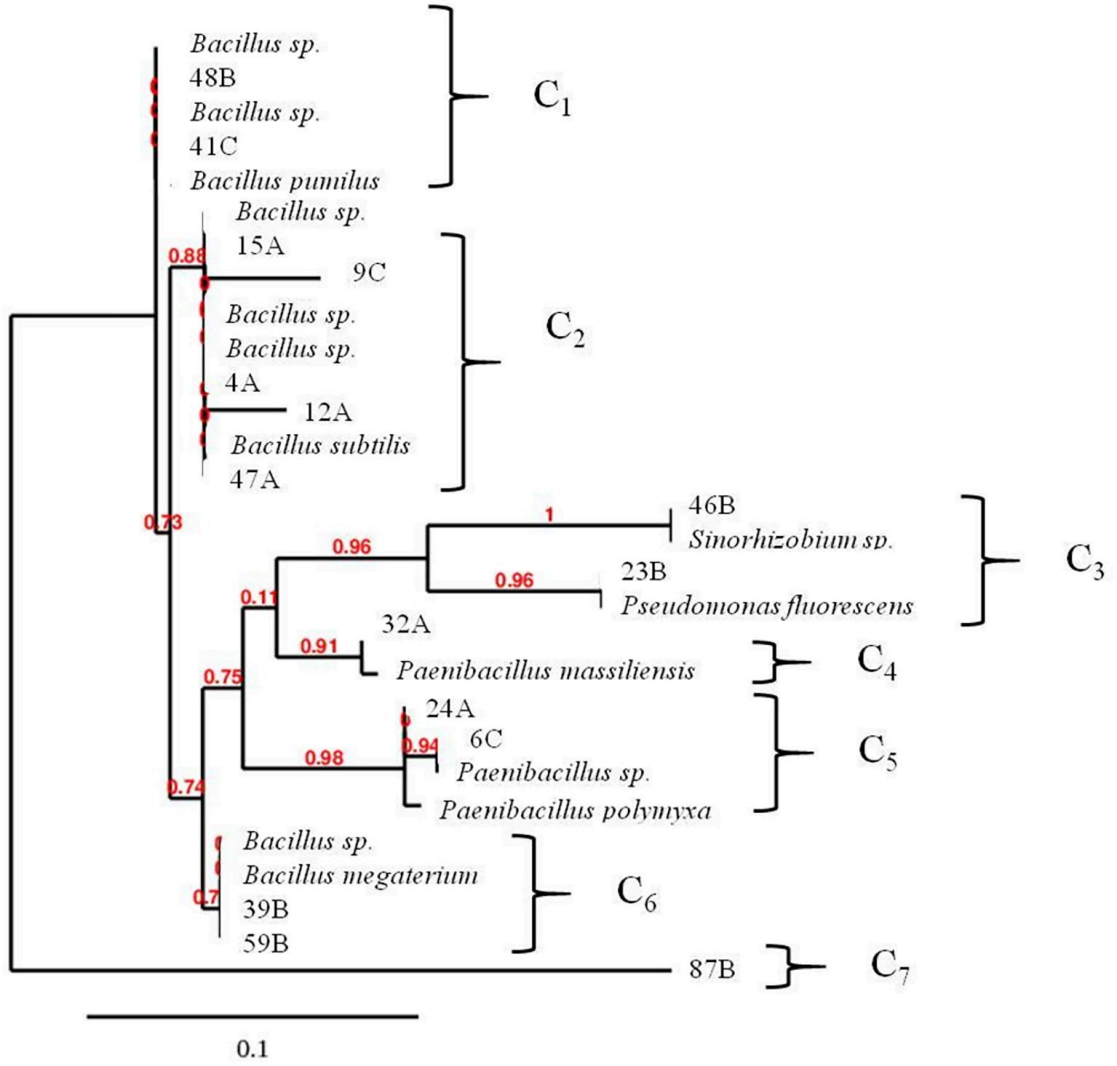
Phylogenetic tree based on 16S rDNA sequence of the 15 bacterial isolates with closely related species of the genus *Bacillus*, *Paenibacillus*, *Sinorhizobium* and *Pseudomonas* using SH-like test with maximum likelihood method. Numbers at nodes indicate percentages of occurrence in 100 bootstrapped trees. The scale bar indicates substitutions per nucleotide position.

A fragment of about 1.5 Kb was obtained after amplification of the 16S rRNA performed on genomic DNA extracted from the fifteen isolated strains with abilities to solubilize the RP with NH_4_^+^ or NO_3_^-^ as the sole N-source. The comparison of sequences with data available from Rpd Taxonomy tool enabled us to identify the isolates at generic or species level with similarities between 100 and 93% (Table 5). The selected isolates were closely related to four specific genera, *Rhizobium* (46B), *Pseudomonas* (23B), *Paenibacillus* (32A, 24A and 6C) and *Bacillus* (12A, 15A, 4A, 59B, 87B, 47A, 9C, 39B, 41C and 48B). All sequences were submitted to NCBI Genbank and accession numbers are given in Table 5. The phylogenetic analysis based on the 16S rRNA gene sequences of the selected isolates and representative species of closely related taxa formed seven clearly distinguishable clusters (C_1_, C_2_, C_3_, C_4_, C_5_, C_6_ and C_7_) (Fig 3). The first cluster (C_1_) was formed by the strains 48B and 41C with a close relationship with genus *Bacillus sp*. The second cluster (C_2_) was also related to *Bacillus sp*. and included five strains (15A, 4A, 47A, 9C, 12A) although the strains 9C and 12A were slightly more distant than the others. The third cluster (C_3_), composed by 46B and 23B, was connected with Gram negative bacteria such as *Sinorhizobium* sp. and *Pseudomonas fluorescens*, respectively. The 32A isolate, grouped in the fourth cluster (C_4_), had notably relationship with *Paenibacillus massiliensis*. The fifth cluster (C_5_), including 24A and 6C strains, was remarkably related with *Paenibacillus sp*. and *Paenibacillus polymyxa*. 39B and 59B isolates were grouped into the sixth cluster (C_6_) with closely relationship with *Bacillus sp*. and *Bacillus megaterium*. Surprisingly, the strain 87B was individually grouped forming the cluster (C_7_) which was related with any species, even if a 94% of identity was found with *Bacillus sp*. (Table 5).

**Table 5:** Identification of the bacterial strains isolated from the soil A, B and C by 16S rRNA sequencing.

Strains	Source of N	*GenBank accession no	16S rDNA identification		
			Closest reference strain	Similarity %	Accession number
46B	NH_4_^+^	MZ723952	*Sinorhizobium sp*.	94%	AJ012210
12A	NH_4_^+^	MZ723957	*Bacillus subtilis*	93%	AJ276351
15A	NH_4_^+^	MZ723963	*Bacillus sp*.	97%	AY160223
4A	NH_4_^+^	MZ723962	*Bacillus sp*.	98%	AF500205
59B	NH_4_^+^	MZ723961	*Bacillus sp*.	97%	AB066347
	NO_3_^-^				
24A	NH_4_^+^	MZ723955	*Paenibacillus polymyxa*	93%	EF532687
87B	NH_4_^+^	MZ723960	*Bacillus sp*.	94%	AY505514
47A	NH_4_^+^	MZ723959	*Bacillus sp*.	98%	AB055850
9C	NH_4_^+^	MZ723966	*Bacillus sp*.	96%	AB188212
6C	NO_3_^-^	MZ723954	*Paenibacillus sp*.	91%	JX266302
39B	NO_3_^-^	MZ723965	*Bacillus megaterium*	94%	AY553114
32A	NO_3_^-^	MZ723956	*Paenibacillus massiliensis*	96%	AY323608
41C	NO_3_^-^	MZ723964	*Bacillus pumilus*	100%	EF197942
23B	NO_3_^-^	MZ723953	*Pseudomonas fluorescens*	94%	DQ916132.1
48B	NO_3_^-^	MZ723958	*Bacillus sp*.	98%	AM944032

*sequences deposited in NCBI

### PCA analysis

**Fig 4:**
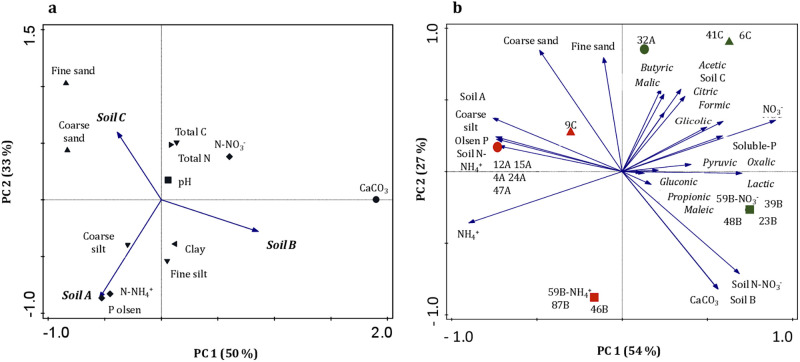
Bi-plot of the distribution. (a) the physical-chemical soil properties in the three sampled sites (A, B and C); (b) the different bacterial strains isolated from the three soils (circles, squares and diamonds indicate strains from soil A, B or C, respectively) with NH_4_^+^ (red color) or NO_3_^-^ (green color) as the sole source of N in relation to the soluble-P content released in the NBRIP liquid media, the production of organic acid (gluconic acid, lactic acid, glycolic acid, acetate, butyrate, formic acid, propionate, pyruvic acid, malic acid, maleic acid, oxalic acid and citric acid) at 21 days of culture and the physical-chemical properties of soil A (Olsen P, NH_4_^+^ content and coarse silt), soil B (CaCO_3_ content and NO_3_^-^ content) and soil C (coarse and fine sand fraction).

A PCA bi-plot was run to analyzed the distribution of the soil physical-chemical properties of the three sites sampled (A, B and C) (Fig 4a). The analysis revealed that soil A was mostly associated with a high content in Olsen P, N-NH_4_^+^ and coarse silt whilst soil B was rather related to a high CaCO_3_ and N-NO_3_^-^ content and soil C was characterized by the presence of coarse and silt sand fractions. Even if soils A and B showed an alkaline pH which differed from soil C (neutral pH values), the PCA analysis did not show any clear relation between pH and type of soil, as similarly was found for the total C and N content. Neither the clay texture nor fine silt was found as properties that intimately characterized the soil A or B.

A set of correlations was performed to evaluate the distribution of bacterial strains according to their abilities to solubilize the rock phosphate under different N sources (NH_4_^+^ or NO_3_^-^) based on the variables measured on the NBRIP liquid media after 21 days of incubation (soluble-P content and organic acids: gluconic, lactic, glycolic, acetic, butyric, formic, propionic, pyruvic, malic, maleic, oxalic and citric acids) and the physical-chemical properties of the three soil sampled (Olsen P, N-NH_4_^+^, N-NO_3_^-^, CaCO_3_, coarse and fine sand fraction). This analysis indicated that 81% of the variability of the data was explained by the two first axes (54% and 27%, respectively) (Fig 4b). The first axis was mainly explained by the N source used to grow the bacteria to assess RP solubilisation (NH_4_^+^ versus NO_3_^-^) and the second axis by the soil texture, especially fine and coarse sand and the CaCO_3_ and N-NO_3_^-^ content. Interestingly, the bacterial strains able to solubilize RP either on NH_4_^+^ or NO_3_^-^ were clearly separated on the bi-plot, clustering with the dominant N source assayed in soil. In addition to the N source, the bacterial strains clustered also along axis 2, forming four groups. For instance, the first group located in the right-upper quadrant was formed by bacterial strains isolated from soil A and C (32A, 41C and 6C) grown with NO_3_^-^ as the sole source of N in the NBRIP liquid media. The strains 32A and 41C were the most efficient in releasing the soluble-P content from RP as the result of the production of organic acids such as: malic, acetic, citric, formic, glycolic, pyruvic and oxalic whereas 6C was the only strains in producing butyric acid. The second group, found in the right-lower quadrant, was only composed by bacterial strains isolated from soil B (59B, 39B, 48B and 23B) still grown with NO_3_^-^ as the sole source of N in the NBRIP liquid media. These bacteria were associated with soil N-NO_3_^-^ and CaCO_3_ contents and the production of gluconic, lactic, propionic and maleic acids. The third group, located in the left-upper quadrant, was composed by strains isolated from soil A (12A, 15A, 4A, 47A, 24A) that were grown on NH_4_^+^ as the sole source of N in the NBRIP liquid media. This group was associated with the Olsen P and N-NH_4_^+^ content of soil A. Finally, in the left-down quadrant was found the fourth cluster composed by bacterial strains isolated from soil B (59B, 87B and 46B) able to solubilize RP with NH4+ and which were associated with CaCO_3_ and NO_3_^-^ contents.

## Discussion

The RP solubilization through PSB has been recently reported as a suitable strategy to cope with the needs of the P requirements of crops [10]. In our study, two hundred and forty-three bacterial strains isolated from three agricultural fields were screened on their abilities to solubilize the RP using the selective NBRIP agarose medium through the *«spot test»*. Our results indicated that only 16% of isolates (forty strains out of two hundred and forty-three) produced a halo of solubilization surrounding the colonies, regardless the N source. This finding was not surprising due to the low solubility and complex chemical structure found in RP compared to other P-sources such as tricalcium phosphate as recently reported [32], which in turn resulted in a less efficient bacterial capacity for dissolving this P-source. Here, we also found that the N-source led to significant differences in the ability to dissolve the RP in the agarose medium with twenty-three isolates showing a halo of degradation with NH_4_^+^ and seventeen positives strains with NO_3_^-^ as N-source. However, except the 59B isolate, none of the strains showed the ability to solubilize the RP with either NH_4_^+^ or NO_3_^-^. It has been reported that the formation of a visible halo/zone on NBRIP agarose medium is a not infallible method to select efficiently the P-solubilizers since they could solubilize important quantities of P in broth despite they did not show a halo zone in NBRIP plate [22]. In fact, in our study, the % of RP-solubilization quantified in liquid media revealed that only nine isolates out of twenty-three (46B, 12A, 15A, 4A, 59B, 24A, 87B, 47A and 9C) were selected as the best RP-solubilizers with NH_4_^+^ as N-source. Meanwhile, seven isolates out of seventeen (6C, 32A, 59B, 39B, 41C, 48B, 23B) showed the greatest % of RP-solubilization with NO_3_^-^. This finding could be explained by the fact that bacterial strains can lose their solubilization phenotype upon repeated sub-culturing, as previously found by [10]. Our results also evidenced that except for 59B isolate, the % of RP-solubilization was higher with NO_3_^-^ than with NH_4_^+^ at 7 days. This observation was surprising since NH_4_^+^ assimilation is usually accompanied of H^+^ extrusion with the subsequent medium acidification, and thus favoring the release of soluble-P from insoluble P-sources [6, 15].

Our second experiment revealed that the highest biomass production found for 46B, 12A, 15A and 4A isolates with NH_4_^+^ as the sole N-source, were not directly related with the highest soluble-P values after 21 days of experiment. [33] proposed that the very effective P-uptake systems of microorganisms would enable the assimilation of P from the solution disturbing the equilibrium between insoluble/soluble-P, thus insoluble-P would be indirectly dissolved by continuously removing of P from the solution. However, a further experiment would be required to verify the amount of soluble-P converted into bacterial biomass. Conversely, the lowest biomass found for 87B isolate resulted in the highest values in soluble-P content, which confirmed a previous evidence showing that bacterial strains were more efficient in dissolving P-sources instead of spending bacterial sources to biomass production [34]. However, the pH drop found for all isolates after 3 days of culture would negatively affect the bacterial growth. In any case, in our study, the pH decline was clearly associated with the RP dissolution since the 87B, 24A and 59B isolates with the lowest pH values, were found to be the most efficient strains increasing the release of the soluble-P from RP between 14 and 21 days of experiment. Meanwhile, the highest pH values detected for 47A isolate were associated with its lesser capacity in dissolving the RP. This was in line with inverse relationship between pH decline of liquid medium and *“in vitro”* RP-solubilization previously reported by [10], indicating that pH plays a major role in the solubilization of the inorganic phosphate. At 21 days, only 39B isolate was found to enhance significantly its growth with a concomitant increase in the soluble-P content with NO_3_^-^ as the sole N-source. This result would be in line with the previous evidences reported by [33, 34] who found that mechanisms of P-solubilization depend on processes associated with microbial biomass production (i.e. NH_4_^+^-assimilation or respiration). Similarly, as it was previously found, the pH drop seemed to be the main responsible of the highest release of soluble-P from RP for 59B, 41C, 32A and 39B isolates at the end of the culture. The use of NH_4_^+^ as a N-source produces acid by either a proton exchange mechanism and/or organic acid secretion with a better P-solubilization [15, 35]. However, our study showed that the assimilation of NO_3_^-^ by bacterial strains resulted in better capabilities to dissolve the RP rather than NH_4_^+^ at 21 days, as the second experiment evidenced but at 7 days. Additionally, the highest levels of soluble-P found for 23B isolate at 3 days suggested that depending on the strain, the mechanisms of RP-solubilization might be also more effective in the short term with NO_3_^-^ as the sole source of N.

The production and secretion of organic acids has been recognized as a major mechanism responsible for releasing soluble-P from RP [9, 10]. Gluconic, formic, citric, oxalic, lactic, succinic, glycolic and acetic acids are among organic acids produced by PSB. In addition to those, pyruvic, malic or fumaric acids were also identified [36]. In our study, regardless the N-source, gluconic, lactic, glycolic, acetic, formic and pyruvic acids were identified as the main organic acids produced by almost all PSB, with the highest concentrations for 12A, 4A, 24A, 87B, 39B, 23B, 59B, 41C, 39B and 32A isolates at 21 days. The production of organic acids during the release of soluble-P from RP, including gluconic, lactic, acetic, oxalic and citric acids among others was demonstrated as directly associated with pH decrease [9, 10, 32]. Our results clearly demonstrated the role of lactic, acetic and formic acids in the RP dissolution for 24A, 87B and 59B isolates when NH_4_^+^ was the sole N-source. Meanwhile, the negative relation between pH and soluble-P content found for 23B, 59B, 41C, 32A and 39B isolates was directly associated with the production of gluconic, glycolic, lactic, acetic, formic, pyruvic, maleic, oxalic and citric acids with NO_3_^-^ as the sole N-source. Interestingly, the concentrations of lactic, acetic and formic acids produced by these isolates were almost 10-fold higher with NO_3_^-^ than with NH_4_^+^, which would be explained by an inhibitory or toxic effect of NH_4_^+^ on the enzymes responsible of organic acid secretion, or uptake of other essential nutrients or altering the electrochemical gradient, as previously suggested [35].

Approximately 86% of the isolates belonged to *Bacillus* and *Paenibacillus* genus, as confirmed the 16S rRNA sequence analysis, which are gram-positive bacteria holding important traits related to the ability to P-solubilization, making them available to be used with agronomical purposes [37, 38]. The isolates 87B, 59B, 39B, 41C, 24A and 32A, which were found to be the most promising strains in dissolving the RP, were closely related to *Bacillus sp*., *Bacillus megaterium*, *Bacillus pumilus*, *Paenibacillus polymyxa* and *Paenibacillus massiliensis*, except for 87B, as revealed the phylogenetic tree. In contrast, only one isolate with the highest ability to secrete lactic acid was identified as *Pseudomonas fluorescens* (23B). In our study, bacterial features involved in the RP dissolution and their co-occurrence in soils would be also influenced by other factors different than N-source, such as physico-chemical properties as others authors suggested [39]. However, this study only revealed that the elevated amounts in N-NO_3_^-^ and CaCO_3_ found in the soil B would be influencing the release of organic acids such as lactic, propionic, gluconic and maleic by the strains: 59B (*Bacillus* sp.), 23B (*Pseudomonas fluorescens*), 39B (*Bacillus megaterium*) and 48B (*Bacillus* sp.). Meanwhile, the Olsen P, N-NH_4_^+^ content and coarse silt in soil A or the sandy texture in soil C seemed not to influence significantly the production of organic acids by neither 12A (*Bacillus subtilis*), 15A (*Bacillus* sp.), 4A (*Bacillus* sp.), 47A (*Bacillus* sp.), 9C (*Bacillus* sp.), 59B (*Bacillus* sp.), 87B (*Bacillus* sp.) nor 46B (*Sinorhizobium* sp.). Interestingly, the PCA analysis revealed that the strains with abilities to dissolving the RP with NH_4_^+^ as source of N were lesser efficient in releasing organic acids, since any positive relation found. Conversely, the strains, 32A (*Paenibacillus massiliensis*), 41C (*Bacillus pumilus*) and 6C (*Paenibacillus sp*.), which were found to assimilate preferentially NO_3_^-^, were positively related with production of butyric, malic, acetic, citric, formic, glycolic, pyruvic and oxalic acids, indicating that the N-source was apparently a key factor driving the bacteria-mediated mechanisms involved in the efficient RP dissolution.

## Conclusions

Our results showed that only 15 strains out of 243 isolates were able to solubilize efficiently the RP with either NH_4_^+^ or NO_3_^-^ as N-source due to the complexity and low solubility of this P-source. Regardless the N-source, the greatest soluble-P content released from RP was detected for 87B, 24A, 59B, 23B, 41C, 32A and 39B isolates, as a consequence of the pH drop. However, 23B, 59B, 41C, 32A and 39B isolates appeared to be the most efficient in dissolving the RP. The strain 59B was the only one with abilities to solubilize the RP with either NH_4_^+^ or NO_3_^-^. Gluconic acid, glycolic acid, lactic acid, acetic acid, formic acid and pyruvic acid were identified in almost all isolates with the highest values with NO_3_^-^ rather than NH_4_^+^. This result evidenced the crucial role of NO_3_^-^ in stimulating the production of organic acids, especially, butyric, malic, acetic, citric, formic, glycolic, pyruvic and oxalic acids secreted by 32A (*Paenibacillus massiliensis*), 41C (*Bacillus pumilus*) and 6C (*Paenibacillus sp*.). The 86% of the isolates were identified as Gram positive bacteria belonging to *Bacillus* sp. and *Paenibacillus* sp. genera, indicating its suitability for agronomical purposes. Other factors different than N-source, but in a lesser extent, would be also involved in the secretion of organic acids such as soil CaCO_3_ and N-NO_3_^-^ content. Our results concluded that the effectiveness of the RP-solubilization would be directly associated with the organic acids production which secretion seemed to be driven by the assimilation of NO_3_^-^ as N-source. Therefore, the N-source might be a key factor to take into consideration during the screening and selection of suitable strains involved in the P-solubilization.

## Supporting information

S1 Table Total contents of elements present in the rock phosphate (RP). Data are given as means ± standard error (n = 3).
(DOCX)

S2 Table The eluent gradient (100 mM KOH) for the program used to determinate the best anion separation. (DOCX)

S3 Table Names of the compounds and concentrations of solutions used to identify and quantify bacterial organic anion release by ion chromatography (HPIC). (DOCX)
