# Supplementary material for: Improved rock phosphate dissolution from organic acids is driven by nitrate assimilation of bacteria isolated from nitrate and CaCO3-rich soil
Source: PLoS One. 2023 Mar 24;18(3):e0283437. doi: 10.1371/journal.pone.0283437 (PMC10038309; doi:10.1371/journal.pone.0283437)
Supplement: S1 Table — Data are given as means ± standard error (n = 3). (DOCX) [file pone.0283437.s001.docx]

S1 Table. Total contents of elements present in the rock phosphate (RP). Data are given as means ± standard error (n = 3).

| Element | Concentration (g kg^-1^) |
| --- | --- |
| Na | 6.1±0.08 |
| K | 0.5±0.03 |
| Ca | 353±6.24 |
| Mg | 2.6±0.01 |
| Fe | 1.3±0.05 |
| Al | 1.9±0.07 |
| P_2_O_5_ | 360±16 |
