# Supplementary material for: Improved rock phosphate dissolution from organic acids is driven by nitrate assimilation of bacteria isolated from nitrate and CaCO3-rich soil
Source: PLoS One. 2023 Mar 24;18(3):e0283437. doi: 10.1371/journal.pone.0283437 (PMC10038309; doi:10.1371/journal.pone.0283437)
Supplement: S2 Table — (DOCX) [file pone.0283437.s002.docx]

S2 Table. The eluent gradient (100 mM KOH) for the program used to determinate the best anion separation.

| Elution time (min) | 100 mM KOH (%) |
| --- | --- |
| 0 | 10 |
| 3 | 30 |
| 4 | 30 |
| 12 | 45 |
| 16 | 70 |
| 20 | 70 |
| 21 | 80 |
| 23 | 80 |
| 24 | 70 |
| 28 | 70 |
| 31 | 10 |
| 35 | 10 |
