# Supplementary material for: Improved rock phosphate dissolution from organic acids is driven by nitrate assimilation of bacteria isolated from nitrate and CaCO3-rich soil
Source: PLoS One. 2023 Mar 24;18(3):e0283437. doi: 10.1371/journal.pone.0283437 (PMC10038309; doi:10.1371/journal.pone.0283437)
Supplement: S3 Table — (DOCX) [file pone.0283437.s003.docx]

S3 Table. Names of the compounds and concentrations of solutions used to identify and quantify bacterial organic anion release by ion chromatography (HPIC).

|  | Concentrations | | |
| --- | --- | --- | --- |
| Compounds | Stock solutions (mM) | RT* determination solutions  (mM) | Calibration solutions (µM)** |
| Gluconic acid | 10 | 3 | 6.25-400 |
| L(+) lactic acid | 20 | 3 | 12.5-800 |
| Glycolic acid | 20 | 3 | 9.37-600 |
| Sodium acetate | 10 | 1 | 9.37-600 |
| Formic acid | 20 | 3 | 12.5-800 |
| Sodium butyrate | 10 | 1 | 9.37-600 |
| Sodium propionate | 10 | 1 | 9.37-600 |
| Pyruvic acid | 10 | 1 | 12.5-800 |
| Glutaric acid | 10 | 1 | 9.37-600 |
| DL-malic acid | 20 | 1 | 6.25-400 |
| Malonic acid | 20 | 3 | 6.25-400 |
| Maleic acid | 10 | 3 | 6.25-400 |
| Oxalic acid | 10 | 3 | 6.25-400 |
| Fumaric acid | 10 | 1 | 9.37-600 |
| Tri-sodium citrate | 10 | 3 | 9.37-600 |

*RT: Retention time; ** For each compound, the calibration curve was built with 7 concentrations distributed between the limit concentrations given in the table.
